# High-throughput sequencing reveals that microRNA-based regulation, cell wall remodeling and phytohormone signaling orchestrate wheat seminal root development

**DOI:** 10.1007/s00425-026-05069-w

**Published:** 2026-07-06

**Authors:** Giorgia Tonielli, Alessia D’Agostino, Gabriele Di Marco, Gerardo Pepe, Chiara Pontecorvi, Anna Fiorillo, Adelaide Teofani, Manuela Helmer-Citterich, Antonella Canini, Angelo Gismondi

**Affiliations:** 1 Laboratary of Molecular Botany, Department of Biology, University of Rome Tor Vergata, Rome, Italy; 2 Laboratary of Bioinformatics, Department of Biology, University of Rome Tor Vergata, Rome, Italy; 3 Laboratary of Plant Physiology, Department of Biology, University of Rome Tor Vergata, Rome, Italy; 4 PhD Program in Molecular and Cellular Biology, Department of Biology, University of Rome Tor Vergata, Rome, Italy

**Keywords:** *Triticum aestivum* L, Seed germination, MiRNomics, Transcriptomics, Root differentiation

## Abstract

**Main conclusion:**

Using a combined RNA and small RNA sequencing approach, this study decodes the precise molecular mechanisms and microRNA-gene networks that govern early seminal root development in wheat. The findings pinpoint specific genetic and hormonal targets that can be leveraged through precision breeding to engineer climate-resilient crops with optimized root architectures.

**Abstract:**

Climate change exerts immense pressure on wheat, threatening both its development and productivity. The transition from dormancy to seedling establishment is a critical yield checkpoint, where seminal roots act as the *hidden architects* of success. Within days of germination, roots must rapidly construct complex systems and adapt to environmental shifts. This early developmental phase determines seedling fate, yet the molecular mechanisms governing it are yet to be fully explored. Thus, in this study, we employed an integrative RNA and small RNA sequencing approach to dissect the regulatory networks governing *Triticum aestivum* seminal root development during the first weeks after seeding. Our work reveals that this stage requires the coordinated action of 385 genes and 12 microRNAs (miRs). Identified as differentially expressed, these molecules orchestrate cell division, metabolic reprogramming, and developmental patterning. Functional enrichment analysis showed that cell wall biosynthesis and remodeling, SNARE-mediated vesicular trafficking, terpenoid metabolism, and phytohormone signaling pathways are dynamically regulated during early root growth. Among all, miR166, miR168, and miR171 emerged as pivotal post-transcriptional regulators. These miRs exhibited expression patterns inversely correlated with their predicted targets, encoding HD-ZIP III transcription factors, spliceosomal kinases, and GRAS like family proteins, which are essential factors for vascular patterning, microRNA biogenesis, and lignin deposition, respectively. Notably, these genetic programs are synchronized with dramatic hormonal recalibration, marking the transition from dormancy to active growth. Beyond advancing our fundamental understanding of root biology, the present findings identify specific molecular targets (i.e., stage-related expressed genes and miRs) that could be manipulated through precision breeding or genome editing to develop wheat varieties with enhanced root systems resilient to environmental changes.

**Graphical abstract:**

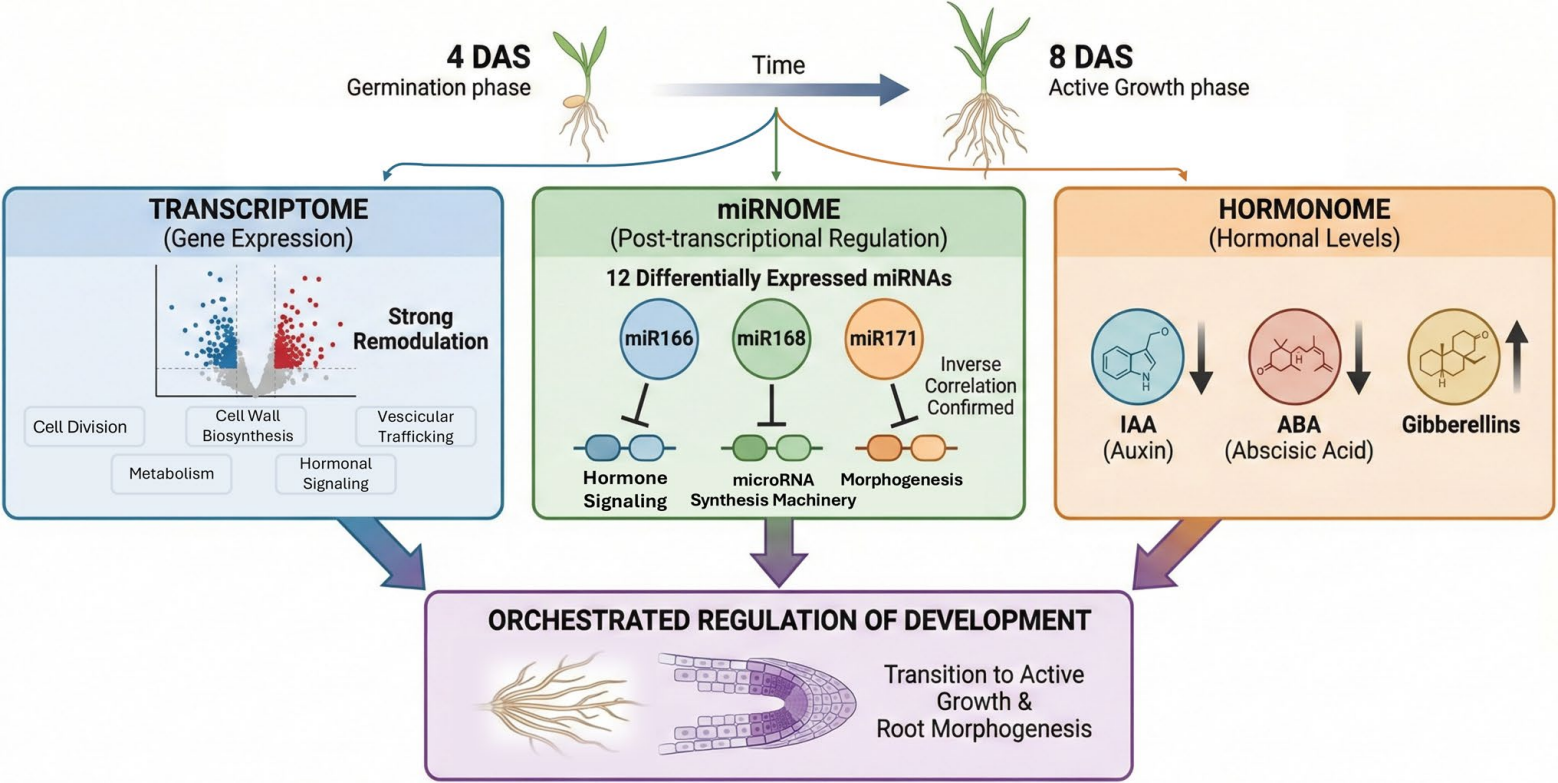

**Supplementary Information:**

The online version contains supplementary material available at 10.1007/s00425-026-05069-w.

## Introduction

Wheat (*Triticum* sp.) is one of the most cultivated crops worldwide and plays a pivotal role in global food security due to its nutritional value. In fact, it provides most of the fibers, minerals, macro- and micro-nutrients and vitamins in the human diet (Tadesse et al. 2019; Igrejas & Branlard 2020). According to the FAO (Food and Agriculture Organization of the United Nations), in 2024, about 700 million tons of wheat were harvested globally, underscoring the importance of this species in the market. However, its economic value also lies in the potential exploitation of agricultural by-products. Indeed, wheat is considered an attractive source of first-generation biofuel thanks to its high starch content which, once converted into sugars via saccharification, can be fermented into ethanol (Cavelius et al. 2023; Taghizadeh-Alisaraei et al. 2023).

Nowadays, 95% of the cultivated wheat is hexaploid bread wheat, while the remaining 5% consists of tetraploid durum wheat (Shewry 2009; FAO 2024). Common bread wheat (*Triticum aestivum* L.) is an annual grass belonging to the Poaceae family (Tegge 1987) that grows preferentially in temperate climates. It can be classified according to its growing season as winter or spring wheat (Fossati & Ingold 2001; Faltermaier et al. 2014). Due to its genetic diversity, there are over 25,000 varieties of wheat adapted to a wide range of environments (Feldman et al. 1995).

Unfortunately, the current grain yield rate will result in insufficient production in the next future due to the increasing worldwide demand of an ever-growing human population and the challenges posed by climate change (Hall & Richards 2013; Hickey et al. 2019). In particular, among all crops, wheat development requires high environmental stability (Yanagi 2024). Indeed, it has been documented how extreme temperature events and fluctuating rainfall patterns affect wheat production (Porter 1999; Ghobadi et al. 2011; Nassar et al. 2013; Farooq et al. 2014; Hatfield & Prueger 2015). Furthermore, global warming contributes to an increased risk of pathologies and pest infestations, affecting both yield and quality (Tian et al. 2019; Bajwa et al. 2020). Therefore, to overcome this issue, in recent years, the attention of scholars has been focused on the *hidden half* of plants (i.e., the root apparatus) (Ober et al. 2021). Roots represent fundamental organs that anchors the plant to the soil, mediate nutrient and water uptake, and act as the primary interface with the environment (Adeleke et al. 2020).

Wheat underground structure is composed of seminal (embryonic) and nodal (post-embryonic) roots, which differ in both anatomy and function during growth (Pigolev et al. 2021). Specifically, primary seminal roots emerge from the coleorhiza within a few days after imbibition and initiate the formation of the root system (Shorinola et al. 2019). While sensitive to exogenous factors, especially in domesticated cultivars, they play a key role in plant growth (Manschadi et al. 2008; Golan et al. 2018; Adeleke et al. 2020). According to this premise, a larger seminal root system may be considered as a favorable agricultural feature promoting crop production and resilience (Pigolev et al. 2021). Breeding programs, based on genetic marker-assisted choices for quantitative and qualitative trait *loci*, have been developed to select future varieties (Adeleke et al. 2020; Ober et al. 2021; Alrajhi et al. 2024). However, these methods may be limited by continuously shifting ecological conditions (López-Bucio et al. 2003; Desnos 2008), thus requiring innovative biotechnological approaches.

Root system adaptation to environmental pressures is driven by gene expression changes at both the transcriptional and post-transcriptional levels (Khan et al. 2011). About the latter, microRNAs (miRNAs; miRs) have been recently proposed as key regulators of root growth (Yan et al. 2022). MiRNAs are small non-coding (20–24 nt) single-stranded RNA molecules able to modulate gene expression, by binding mRNA targets through the RISC (RNA-Induced Silencing Complex) and mediating their cleavage or translation inhibition (Kidner & Martienssen 2005). In plants, miRNA expression is tissue/cell type- and time-specific, being involved in several key biological processes, including embryonic, primary, lateral and adventitious root development and vascular differentiation (Chen 2005; Khan et al. 2011; Yan et al. 2022). Using high-throughput sequencing analysis, previous researchers have identified cellular pathways involved in wheat root responses to abiotic and biotic stresses (Yao et al. 2007; Gupta et al. 2014; Wang et al. 2014; Eren et al. 2015; Derakhshani et al. 2020; Xu et al. 2022; Xi et al. 2023). However, the specific molecular mechanisms triggered in wheat seminal roots during the first week of growth under physiological conditions remain largely undocumented. To address this scientific gap, this study aimed to investigate the transcriptomic and miRNomic profile of *T. aestivum* seminal roots during early development, the most critical period for seedling establishment. Our results demonstrate the modulation of pathways linked to cell wall production and phytohormone signaling mediated by specific miRNAs (e.g., miR166, miR168 and miR171). Therefore, our evidence sheds light on the biological processes triggered in wheat seminal roots, fundamental structures for crop stabilization and development, also evidencing the existence of finely regulated gene control systems at both transcriptional and post-transcriptional levels.

## Materials and methods

### Plant material and growth conditions

*Triticum aestivum* L. subsp*. aestivum* seeds (Altamira variety) were kindly provided and genetically guaranteed by Limagrain Italia Spa (Fidenza, Parma, Italy). Seeds were sterilized with 3% sodium hypochlorite (diluted in bidistilled water; 50:50, *v/v*), rinsed three times with bidistilled water, and left in imbibition for 1 h in dark. The sterilized seeds were planted in pots, on filter papers previously hydrated with sterilized bidistilled water, and left in the dark for 48 h. Then, pots were positioned in a phytotron for 12 days after seeding (DAS) under controlled conditions: temperature of 22 °C, photoperiod of 14 h light/10 h dark, homogeneous light intensity of 120 μmol m^−2^ s^−1^. During this period, seedlings were irrigated every two days, saturating the filter papers with water. At the selected stages of growth (4, 8, and 12 DAS), plant material was harvested, ground to a fine powder in liquid nitrogen using mortar and pestle and stored at − 80 °C until use.

### RNA extraction and sequencing

For RNA extraction, WizPrep Plant RNA Mini Kit (Wizbiosolutions, Loco Hills, New Mexico) was used according to the manufacturer’s instruction. Briefly, 100 mg of powder from wheat roots was resuspended with 500 µL of lysis buffer and purified by centrifugation. Then, the RNA present in the supernatant was bound to a filter column and subjected to several washing steps. At the end, RNA was eluted and its concentration and purity estimated by spectrophotometric analysis (NanoDrop 2000, Termo-Fischer Scientific, USA). RNA quality was assessed by a TapeStation 4200 system (Agilent Technologies, Santa Clara, CA), before library preparation via Illumina Stranded mRNA Prep kit (Illumina, San Diego, CA). Libraries were checked and quantified using the Tape Station 4200 (Agilent Technologies) and Qubit Fluorometer (Invitrogen Co., Carlsbad, CA) instruments and then pooled together such that each index-tagged sample was present in equimolar amounts. Finally, next-generation sequencing (NGS) analysis was carried out using Illumina Novaseq6000 System (2 × 75 paired-end format; Illumina, San Diego, CA, USA). At least 80% of bases resulted characterized by a quality score of 30 or higher.

### Transcriptome analysis

Sequencing quality assessment and pre-processing were conducted using FastP (version 0.23.2) (Chen et al. 2018). Reads passing quality filters were aligned to wheat (*T. aestivum*) reference genome (IWGSC RefSeq v57), obtained from the Ensembl Plants public database. Read alignment was performed using Bowtie2 (version 2.2.5) (Langmead and Salzberg 2012), while gene-level quantification of the aligned reads was carried out with HTSeq (version 2.0.4) (Putri et al. 2022). Gene expression raw counts were normalized to Counts Per Million (CPM) to account for variations in sequencing depth and library size, enabling comparisons across samples. Differential expression analysis between samples (e.g., Nr1 *vs* Nr2) was performed using the CPM values. For each gene, the fold change was calculated as the ratio of expression levels in Nr1 relative to Nr2, capturing both direction and magnitude of the expression change. To assess the statistical significance of the expression differences, a *Z*-score value and a corresponding *p*-value were calculated for each gene, quantifying the extent to which observed expression deviated from expectations under the null hypothesis of no differential expression. To correct for multiple hypothesis testing and control the false discovery rate (FDR), the Benjamini–Hochberg procedure was applied to the *p*-values (Benjamini & Hochberg 1995). Genes with an adjusted *p*-value showing a FDR ≤ 0.05 were considered significantly differentially expressed. Functional enrichment analysis was performed on two sets of differentially expressed genes (59 and 385, selected by varying the stringency of the analysis as reported above using *Z*-score from − 2 to 2 and adj *p*-value ≤ 0.05, respectively) using PANTHER 18.0 (Thomas et al. 2021), Metascape (Zhou et al. 2019) and SRplot (Tang et al. 2023), categorizing genes based on molecular function, biological process, and pathway annotations. To enable cross-species functional annotation, wheat IWGSC RefSeq gene identifiers were mapped to *Arabidopsis thaliana* (L.) Heynh. Gene identifiers using the g:Orth tool available on g:Profiler (Kolberg et al. 2023).

### RNA retrotranscription and quantitative real time PCR assays

Total cDNA was synthetized from RNA previously isolated using WizScript cDNA synthesis Kit (Wizbiosolutions, Loco Hills, New Mexico) according to the manufacturer’s instruction. Each RT-qPCR reaction, instead, was performed by mixing 20 µg of cDNA, 2X Fast q-PCR Master mix (Syber Green with ROX, SMOBiO, Hsinchu City, Taiwan), and 1 µL (5 µM) of each primer (Supplemental Material 1 – Table S1). Amplifications were performed using a StepOnePlus Real-Time PCR System (Applied Biosystems) set as reported: (i) initial denaturation at 95 °C for 10 min; (ii) 65 cycles of denaturation at 95 °C for 20 s and primer annealing at 59 °C for 30 s; (iii) production of dissociation curve, from 50 to 95 °C (rate: 0.3 °C every 15 s). The amount of mRNA for each gene was quantified using the 2^−ΔΔCt^ formula, where the threshold cycle (Ct) of the target gene detected in the treated sample was normalized for the internal reference gene (*eIF4A*, ΔCt) and for the respective value observed in control samples (ΔΔCt), considered as unit.

### MicroRNA extraction and sequencing

MicroRNAs were isolated from powder of wheat roots, using miRPremier microRNA Isolation Kit (Sigma-Aldrich, St. Louis, USA). In brief, 100 mg of plant material was resuspended with 750 µL of lysis solution, purified by filter columns, subjected to several washing steps, and finally to microRNA elution. Concentration and purity of the extracts were estimated by spectrophotometric analysis (NanoDrop 2000, Termo-Fischer Scientific, USA). Then, the samples were subjected to NGS analysis: libraries were prepared using NEX SmallRNA Seq v3 (Perkin Elmer, Shelton, Connecticut, U.S.) and quantified using Tape Station 4200 (Agilent Technologies) and Qubit Fluorometer (Invitrogen Co., Carlsbad, CA) instruments. NGS was carried out by an Illumina Novaseq6000 System (1 × 75 single-end format; Illumina, San Diego, CA, USA), pulling together all libraries such that each index-tagged sample was present in equimolar amounts. At least 80% of bases resulted characterized by a quality score of 30 or higher.

### MiRNome analysis

Raw data quality from small RNA sequencing was evaluated using FastQC (v0.12.1) (Andrews 2010). Based on the FastQC reports, the reads were further processed by Cutadapt (v4.7) (Martin 2011). In particular, the Illumina Small RNA 3’ Adapter was removed, low-quality bases at 3’ end (for Illumina reads, quality is high at the beginning, but degrades toward the 3’ end) were trimmed and flanking N bases from each read was eliminated, employing parameters like ‘-a TGGAATTCTCGGGTGCCAAGG’, ‘-q 30’, ‘–trim-n’. Finally, only reads ranging from 21 to 25 nucleotides were kept, to ensure that only miRNA-derived reads were retained. Previously filtered reads were aligned to the miRBase database (Release 22.1) (Kozomara et al. 2019). Given that miRBase includes identical miRNAs from various species, reads were selectively aligned to miRNAs classified under Viridiplantae, to mitigate alignment noise. To address the residual data redundancy, miRNA sequences were clustered based on a 90% sequence similarity threshold and a representative miRNA for each cluster was determined using CD-HIT (v4.8.1) (Li & Godzik 2006). In detail, sequences were first sorted in a descending manner according to their length and then the longest sequences were taken as the representative members for each cluster. Thus, high-quality reads were aligned to the Viridiplantae-specific subset of miRBase utilizing the BBMap short-read aligner tool (Bushnell 2014). Alignments were stored in SAM files and the mapped read-segments were retrieved using samtools idxstats (Li et al. 2009). At this stage, a custom Python script was used to assign to each representative miRNA sequence the sum of all sequences counts within its cluster. Subsequently, low-count miRNAs were filtered out and only those with a minimum of 10 counts in at least 50% of the samples were maintained, obtaining a total of 61 miRNAs. After that, the count data were normalized to account for differences in library size. To do that, mapped reads (that is the number of raw reads mapped to a miRNA) were scaled by the library size in each sample and multiplied by 10^6^. Differential expression analysis of miRNAs was also conducted across all samples by calculating fold change as follows: log_2_ fold change = log_2_ (B condition/A condition). Differentially expressed miRNAs (DEMs) were those having a *Z*-score >/=|1| for the comparison 8 DAS vs. 4 DAS. The sequences of DEMs represented the inputs for psRNATarget software (Dai et al. 2018), used to identify their putative *T. aestivum* mRNA targets (selecting the reference “*T. aestivum* cDNA library from Ensemble plants, release 43”). To understand the functions of the genes targeted by DEMs, a GO enrichment analysis was performed by Panther (Mi & Thomas 2009) and also by Metascape (Zhou et al. 2019). Given that Metascape does not support *T. aestivum* organism, orthologous genes in *A. thaliana* were searched with g: Orth, a gprofiler tool (Kolberg et al. 2023).

### MicroRNA retrotranscription and RT-qPCR

The cDNA relative to miRNA samples was synthesized using miRCURY LNA RT Kit (QUIAGEN, Hilden, Germany) according to the manufactures’ guidelines. On the other hand, RT-qPCR assays were carried out using miRNA cDNA as template and following the protocol reported in detail in Gismondi et al. (2017). During the experiments, a StepOnePlus Real-Time PCR System (Applied Biosystems) was employed, together with 2X Fast q-PCR Master mix reagent (Syber, ROX) (SMOBiO, Hsinchu City, Taiwan). In detail, the presence of UniSp6 (considered as positive control to check the validity of the system, EXIQON), miR166 (miRBase accession number MIMAT0043967, EXIQON), miR168 (MIMAT0043887, EXIQON), miR171 (MIMAT0037396, EXIQON), and plant 5S rRNA (considered as internal controls; *A. thaliana* GenBank: AB073495.1, EXIQON) was investigated.

### Hormone extraction and quantitation

The extraction of hormones from wheat roots was carried out according to Trupiano et al. (2012). In detail, 500 mg of frozen powdered sample was resuspended with 1250 mL of methanol and then centrifuged at 16.000 g for 10 min at 4 °C. Supernatant was recovered and concentrated under vacuum (Eppendorf AG 22331 Hamburg, Concentration Plus). Then, samples were re-suspended with pure water adjusted to pH 9 and an equal volume of ethyl acetate. Aqueous and organic phases were separated by centrifugation at 16.000 g for 2 min. The lower aqueous phase was collected and acidified at pH 3 to maintain the hormones in their protonated form. After further centrifugation, the upper phase was recovered and completely dried under vacuum. The samples were re-suspended in 30 µL of methanol and analyzed by reversed-phase high-performance liquid chromatography according to Manai et al. (2024). In particular, an LC-20 Prominence HPLC system, associated with a diode array detector (Shimadzu, Kyoto, JP) and a Gemini–NX C18 column (250 × 4.5 mm, 5 µm particle size) (Phenomenex), was used. Results were expressed as ng of phytohormone per mg of plant material.

### Statistics

For transcriptomic (paired-end RNA-seq) and small RNA (single-end miRNA-seq) sequencing, a robust strategy was employed to capture a representative profile for each condition (4 and 8 DAS). Specifically, total RNA/miRNA was purified in triplicate; each replicate derived from 30 specimens grown in an independent biological batch, totaling 90 seedlings per experimental point. The pool of these highly representative biological extracts was subjected to library construction, minimizing individual stochastic variation and ensuring a comprehensive overview of the developmental stages. qPCR assays and hormone profiling were conducted using three independent biological replicates. Technical consistency was ensured by performing the measurements at least in triplicate. Data are presented as mean ± standard deviation. Statistical significance was assessed via one-way analysis of variance (ANOVA), followed by Fisher’s least significant difference (LSD) post hoc test. Differences were considered statistically significant at a *p*-value < 0.05 and indicated with distinct letters. All statistical procedures were performed using Microsoft Excel.

## Results and discussion

The early growth stage is a critical phase of plant development. Optimal seed germination and vigor are necessary for a crop to establish itself firmly and reach maturity (Ashraf & Abu‐Shakra 1978). In wheat, for example, seedling survival rate and vegetative growth directly influence final field yield. This pivotal phase is initiated by seed imbibition, followed by the remobilization of storage resources to support coleoptile and root growth (Yu et al. 2014). Then, wheat seminal roots play a crucial role during embryogenesis, supplying water and nutrients, while controlling their absorption rate. Furthermore, a well-established root system is required to correctly develop crop aerial portion and establish the first interface with the external environment (e.g., rhizobia, soil components, and stressors) (Huang et al. 1991; Harley 2013).

**Fig. 1:**
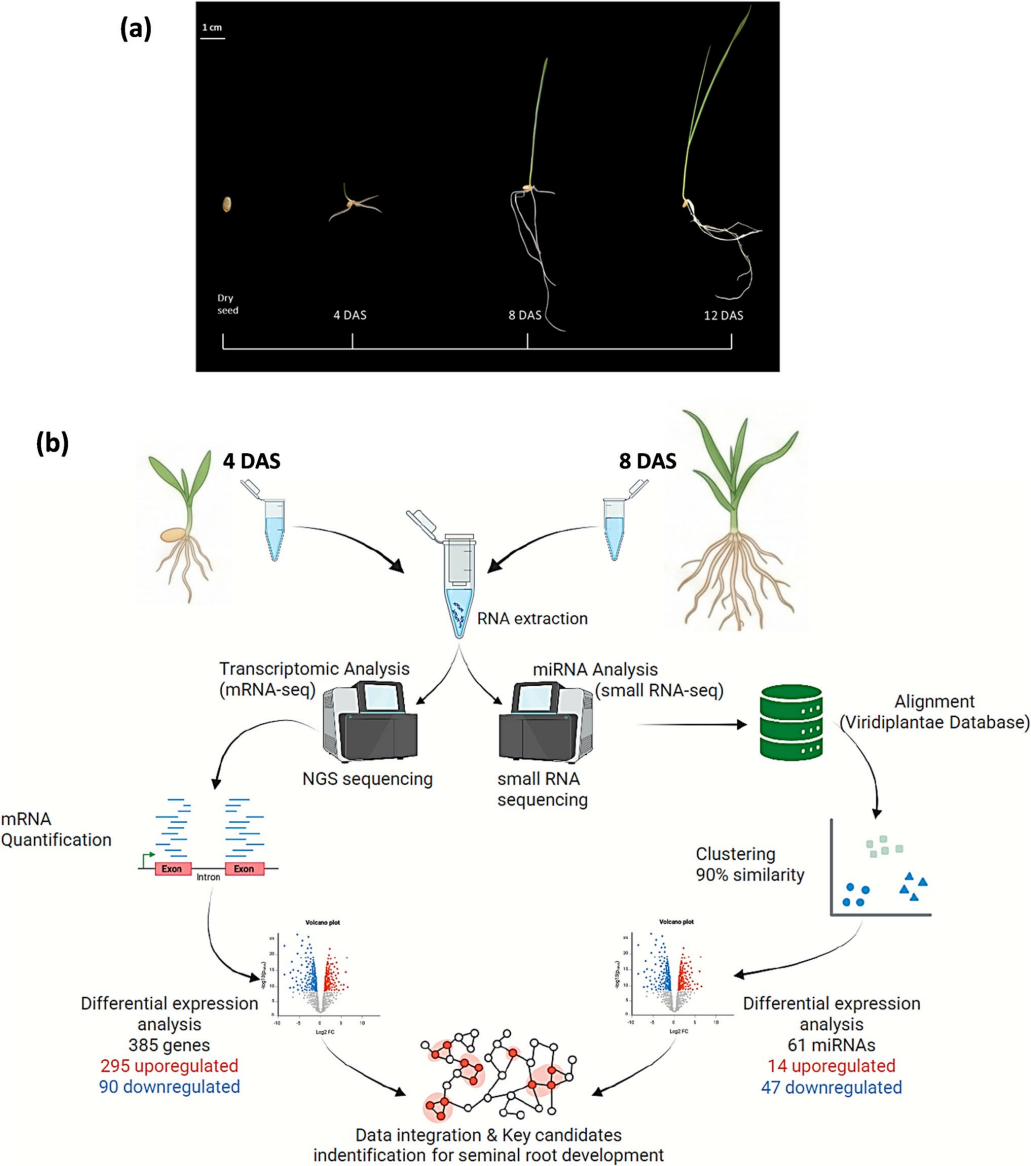
Plant material. **a** Representative image of *T. aestivum* (Altamira variety) seedling at 4, 8 and 12 Days After Seeding (DAS). **b** Workflow followed to carry out the two omics approaches applied to plant material

This evidence suggests that understanding the complexity of root development is key to unlocking selection programs and biotechnological strategies for obtaining high-yielding and climate-resilient plant varieties. Thus, in this study, gene expression analysis of wheat seminal roots was carried out through two different omics techniques to investigate the main cellular and molecular mechanisms underlying root system development during the first weeks following germination (Fig. 1 a, b).

### Transcriptomics provides evidence of cell wall and hormone signaling modulation

To obtain a general overview of the specific genes potentially involved in seminal root development in wheat, transcriptomic analysis was performed on samples collected at 4 and 8 DAS.

**Fig. 2:**
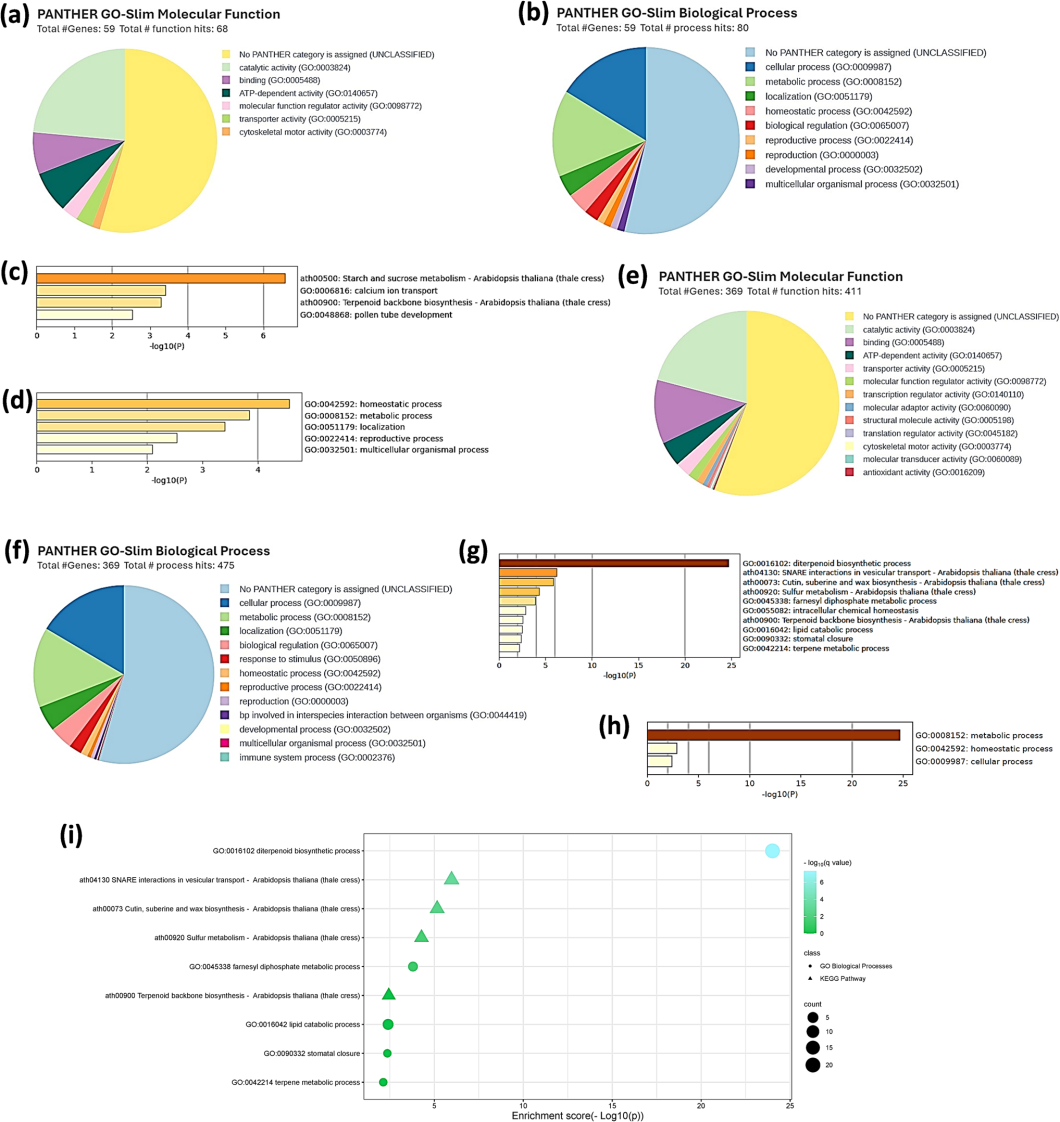
Gene Ontology (GO) enrichment analysis of transcriptomics data. **a** PANTHER pie chart of enriched terms in the molecular functions category obtained from the list of 59 genes. **b** PANTHER pie chart of enriched terms in the biological process category from the list of 59 genes. **c** Metascape bar graph of top level GO enriched terms across the input obtained from the list of 59 genes (bar color indicates the *p*-value). **d** Metascape bar graph of parent level GO enriched terms across the input obtained from the list of 59 genes (bar color indicates the *p*-value). **e** PANTHER pie chart of enriched terms in the molecular functions category obtained from of the list of 385 genes. **f** PANTHER pie chart of enriched terms in the biological process category from the list of 385 genes. **g** Metascape bar graph of top level GO enriched terms across the input obtained from the list of 385 genes (bar color indicates the *p*-value). **h** Metascape bar graph of parent level GO enriched terms across the input obtained from the list of 385 genes (bar color indicates the *p*-value). **i** SRplot showing GO/KEGG terms resulting using as input the upregulated genes from the list of 385 (for each term, -log_10_(*p*-value) is represented by colors, while gene counts by bubble size)

RNA next-generation sequencing approach detected almost 40 million reads across all the samples. Bioinformatic analysis provided a list of 59 genes which resulted significantly modulated between 4 and 8 DAS (Supplemental Material – Table S2). To gain insights into the molecular functions (MFs) related to these genes and identify the biological processes (BPs) in which they are involved, gene ontology (GO) enrichment analysis was conducted using PANTHER. The most enriched GO terms involved for MFs were catalytic activity, binding, ATP-dependent activity, regulator and transporter activity, and cytoskeletal motor activity (Fig. 2a). Meanwhile, the resulting BP-GO terms were cellular, metabolic, homeostatic, and developmental processes, as well as biological regulation (Fig. 2b). To obtain further information, Metascape software was also applied to the same dataset. This web tool corroborated the evidence from PANTHER but also suggested novel GO/KEGG terms, such as starch and sucrose metabolism, calcium ion transport, and terpenoids backbone biosynthesis pathway, indicating their regulation during wheat seminal root development (Fig. 2c, d).

Considering that the first list of genes obtained by NGS analysis consisted only of upregulated transcripts, a less stringent filter was applied (see Material and Methods for details), generating from the same counts a second group of genes which was composed of 385 genes, with 295 upregulated and 90 downregulated (Supplemental Material – Table S2). Even in this case, GO enrichment analysis was conducted. As expected, PANTHER evidenced more MF-GO terms than those described in the previous prediction, including novel elements like transcription and translation regulator activity, molecular adaptor, transducer activity, and antioxidant activity (Fig. 2e). Similarly, for the BPs category, additional GO terms were registered, such as response to *stimulus* and immune system process (Fig. 2f). Using the same second list of genes, Metascape also expanded the number of pathways potentially associated with wheat root growth. In particular, terms like diterpenoid biosynthetic process, SNARE interaction in vesicular transport, cutin, suberin and wax biosynthesis, intracellular chemical homeostasis and lipid catabolic process were found (Fig. 2g, h). Lastly, a bubble plot was created by SRplot software using only the list of up-regulated genes as input. The data, shown in Fig. 2i, corroborated the preceding analyses, suggesting that terpene metabolism and cell wall/membrane reshaping were the most prominent functions occurring in the samples. These results could be explained by considering that during the first weeks after the imbibition, intense sprout growth due to continuous cell divisions is observed. Indeed, root elongation and development are mainly due to a fine-tuned balance between cell proliferation in the meristematic region and cellular differentiation in the elongation zone, where cells cease mitosis and start to specialize (Kirkham 2014). In this context, transcriptomic data showed the activation of the *TraesCS5B02G125300* gene, predicted to encode an HVA22 like protein that is related to seedling germination. Indeed, HVA22 is an abscisic acid (ABA)-induced plant protein, initially isolated from barley (*Hordeum vulgare* L.) aleurone cells, whose accumulation is linked to nutrient mobilization, seed germination and growth (Shen et al. 2001; Guo & David 2008). Seed germination and embryonic root formation are associated with fundamental changes, such as the synthesis and redistribution of subcellular organelles and specific molecules but also the switch from anaerobic to aerobic metabolism, resulting in cell differentiation (Cervantes 2006). It is plausible to hypothesize how a boost of energy is required at these phases, in particular the up regulation of starch and sucrose metabolism and cellular respiration, as confirmed by the results reported in Fig. 2 (e.g., c and d). In fact, transcriptomic data showed the over-expression of *TraesCS1A02G342500* and *TraesCS1D02G344600*, which are predicted to be hexokinases (i.e., HXK2, HXK1, HKL3), and *TraesCS4B02G211200*, which encodes a putative enolase (i.e., DUF3527). In addition, the increased transcription of the Cytochrome B6f gene (*TraesCSU02G053500*), forming a plastocyanin oxidoreductase complex that catalyzes the quinol oxidation step in oxygenic photosynthesis (Cramer et al. 1994), and of the photosystem II reaction center PsbP family protein gene (*TraesCS5B02G093100*), interacting functionally and structurally with PsbQ within the PSII complex (Ifuku et al. 2005), suggested a higher rate of aerobic metabolism. The hypothesis of increased cell division is also backed by the upregulation of different proteins associated with Mitogen-activated kinase (MAPK) cascades, in particular mitogen-activated protein kinase kinase kinase 3 (*TraesCS4A02G211600*) and MAP kinase 20 (*TraesCS3D02G225600*). The MAPK signaling network controls the plant intracellular response to environmental and developmental *stimuli*, activating several downstream targets. The canonical cascade starts via successive phosphorylation steps mediated by an upstream MAPK kinase kinase (MAPKKK or MKKK) that activates a MAPK kinase (MAPKK or MKK). This phenomenon has been recorded in root cells by transcriptomic studies which have identified MAPK gene expression, especially MAPKKK3 and MAPK20, during defense and division processes (Wang et al. 2022a, b; Zhang & Zhang 2022; Zhang et al. 2024). Furthermore, the transcript levels of enzymes related to the redox equilibrium appeared increased from 4 to 8 DAS: Cytochrome B5 (*TraesCS1D02G184800*) and Cytochrome P450 (*TraesCS4A02G073700*). Cytochrome B5 works as an electron donor for cytochrome P450 but also takes part in fatty acid biosynthesis and modification (e.g., elongation, desaturation and hydroxylation) (Liu 2022). In this regard, SRplot functional analysis highlighted an enrichment of lipid catabolic processes (Fig. 2g, i). Fatty acid production and degradation are crucial during the seedling establishment stage. Seedling growth requires lipid mobilization since this class of substances represents a source of energy and substrates (Cai et al. 2020; Koley et al. 2025). In accordance with this, an upregulation of a thioesterase superfamily protein transcript (*TraesCS5D02G037100*), which mediates the hydrolysis of acyl-coenzyme A (Caswell et al. 2022), and a 3-hydroxyacyl-CoA dehydrogenase family protein transcript (*TraesCS3B02G357200*), which catalyzes the third step of the fatty acid *β*-oxidation process (Feher 2012), was registered. The production of fatty acids and their derivatives is important considering that they are energy storage molecules and key components of plasma membranes but also regulators of plant immune response, participating in the suberin deposition pathway (Domergue et al. 2010; Xiao et al. 2022)*.* Cytochromes P450, on the other hand, participate in the catabolism of different phytohormones but also play a role in DNA repair and histone demethylation (Farrow & Facchini 2014; Liu 2022), which are functions highly requested in maintaining cell division during tissue elongation (Xu et al. 2022). Similarly, other factors associated with the same process, like DEAD/DEAH box helicases, chaperons, DnaJ-domain superfamily proteins, RNA-binding (RRM/RBD/RNP motifs) family proteins and mRNA splicing factor snRNPs (*TraesCS6A02G312100*, *TraesCS4D02G013000*, *TraesCS4A02G203400*, and *TraesCS6B02G292900*, *TraesCS4D02G072700*), presented significant expression changes.

Function enrichment analysis also showed the dominance of various genes involved in sulfur metabolism (Fig. 2 g, i). Sulfur is one of the essential components of a wide range of compounds, including amino acids, vitamins and enzyme (as cofactors). The uptake of its anionic form (SO_4_^2−^) from the soil is mediated by several high or low- affinity sulfate transporters. Regardless, its correct absorption is required for an adequate growth and development (Narayan et al. 2023). For instance, cysteine is one of the main organic products generated from sulfate; it derives from methionine and is important for the generation of glutathione and protein disulfide bonds (Li et al. 2020a, b). After root emergence, the demand for sulfur increases in sprouts; thus, the storage proteins present in the endosperm are degraded to provide new amino acids for protein synthesis and cellular metabolism (Sheoran et al. 2005; Yang et al. 2007). In particular, sulfur amino acids have a large influence on germination and embryonic root growth (Rajjou et al. 2012) as demonstrated by the fact that the inhibition of methionine biosynthetic enzymes leads to a delay in germination (Gallardo et al. 2002b).

Connected to cell division, the evidence of cellular rearrangements and cytoskeletal motor activities also resulted from transcriptomics. Indeed, an increased expression of formin genes (*TraesCS2A02G214500*) and the annexin 5 gene (i.e., *TraesCS3D02G479100*) was found. These two proteins are regulators of cytoskeletal dynamics and cell elongation; in particular, formin proteins work as cytoskeleton–plasma membrane–cell wall linkages, whereas annexins are closely related to the regulation of plant growth and response to environmental *stimuli* in response to Ca^2+^ and ROS accumulation (Grunt et al. 2008; Baucher et al. 2012). In this respect, Ca^2+^ is known to be an essential element as well as a secondary messenger in plant organisms; low levels of this macronutrient in sprouts led to shorter roots, suggesting its importance in this process (Simon 1978; Hepler 2005). In fact, localized influxes of this ion are necessary to provide polarization to cell growth, induce cytoskeletal motility and organization, and regulate microtubule depolymerization during mitosis (Robinson & Cone 1980; Zhang et al. 1992). In line with this evidence, from 4 to 8 DAS, in *T. aestivum* embryonic roots, many transcripts encoding calcium-associated proteins, like Ca^2+^–ATPase or calcium-binding EF-hand family proteins (e.g., *TraesCS4A02G103000*, *TraesCS5B02G276500*, *TraesCS5A02G136100*), appeared upregulated. This type of regulation could be associated with tissue plasticity, highly required during root development. Indeed, while it is possible to observe an upregulation of calcium-associated proteins which determine structural rigidity (Burstrom 1968), an increased expression of transcripts encoding for hydrolases (e.g., glycosyl hydrolase, *TraesCS1D02G074700*; alpha/beta-Hydrolases superfamily protein, *TraesCS3D02G222200*) and plant invertase/pectin methyl esterase inhibitor superfamily proteins (*TraesCS2A02G109600*), which are associated to cell wall degradation and elasticity (Grandis et al. 2019; Coculo & Lionetti 2022), could also be appreciated. In addition, the accumulation of WRKY family transcription factor transcripts (*TraesCS5A02G156700, TraesCS5D02G162100, TraesCS5B02G154900, TraesCS2D02G198100, TraesCS6D02G136200*) suggested a transcriptional regulation of lignin metabolism during root development. Indeed, lignin biosynthesis is finely regulated by many transcription factors, like MYB and NAC, which are activated upstream by WRKY and other proteins (Dong & Lin 2021). More in detail, WRKY factors bind specific W-box sequences in the promoter region of their target genes, mainly involved in plant growth, immune response, and hormone pathways, and promote their expression (Eulgem, & Somssich 2007; Ma & Hu 2024).

Cell wall structure and its macromolecular composition are pivotal for sprout development, signal transduction, and disease resistance. Thus, biosynthesis and deposition of cell wall polymers need to be tightly controlled during wheat germination. In this context, the endomembrane system plays a crucial role in moving polysaccharides, along with the enzymes responsible for their synthesis and modification, as well as glycoproteins, by means of vesicle-mediated transport pathways (Sinclair et al. 2018). The membrane trafficking scheme connects membrane-bounded organelles, such as the endoplasmic reticulum (ER), Golgi apparatus, endosomes, and vacuoles, while coordinating the transport of cell wall components through ER and Golgi (Kim & Brandizzi 2014). This network is finely tuned due to its function not only in fundamental cellular processes but also for more complex physiological processes, including plant development and adaptation to environmental *stimuli* (Surpin, & Raikhel 2004; Uemura & Ueda 2014). Based on this premise, it is important to underline that, during the first week of embryonic root growth, transcriptomic data showed significant changes for SNARE mRNAs (Fig. 2g, i), encoding membrane fusion regulatory proteins (Bassham & Blatt 2018). In particular, some of these transcripts were found upregulated (e.g., SEC22; *TraesCS3B02G184700*), while others were downregulated (e.g., SYP51/52; *TraesCSU02G121500*). This evidence could be linked to the different functions performed by the respective proteins. For instance, SEC22 is involved in anterograde transport from the ER to the Golgi and its *loss-of-function* in *A. thaliana sec22* mutants leads to a loss of Golgi integrity and defects in plant development (Chatre et al. 2005; El‐Kasmi et al. 2011). On the other hand, SYP51/52 tonoplast proteins have a role in vacuole sorting and De Benedictis and colleagues (2013) have showed in *A. thaliana* also a potential i-SNARE (interfering SNAREs) function for them, which is their ability to prevent heterotypic fusion when SNARE complexes on pre-vacuoles are saturated.

Lastly, the diterpenoids biosynthetic process, or more in generally terpenoid production, appeared to be highly promoted during wheat root growth (Fig. 2c, g, i). Terpenes and terpenoids are one of the main classes of phytochemicals carrying out a great variety of functions in plants, during growth and development but also in response to environmental pressures (Cheng et al. 2007; Zhang et al. 2011; Tholl 2015). In this group of metabolites also, isoprenoid phytohormones, such as abscisic acid, brassinosteroids, gibberellins, and strigolactones, can be included (Bajguz, & Piotrowska-Niczyporuk 2023). They are important regulators of different aspects of plant life, including seed germination and root growth (Garay‐Arro et al. 2012), making the detection of the transcripts related to their synthesis expected among the present NGS data.

### MicroRNA expression profile suggests the existence of gene regulation mechanisms linked to morphogenesis, tissue differentiation and hormones

**Fig. 3:**
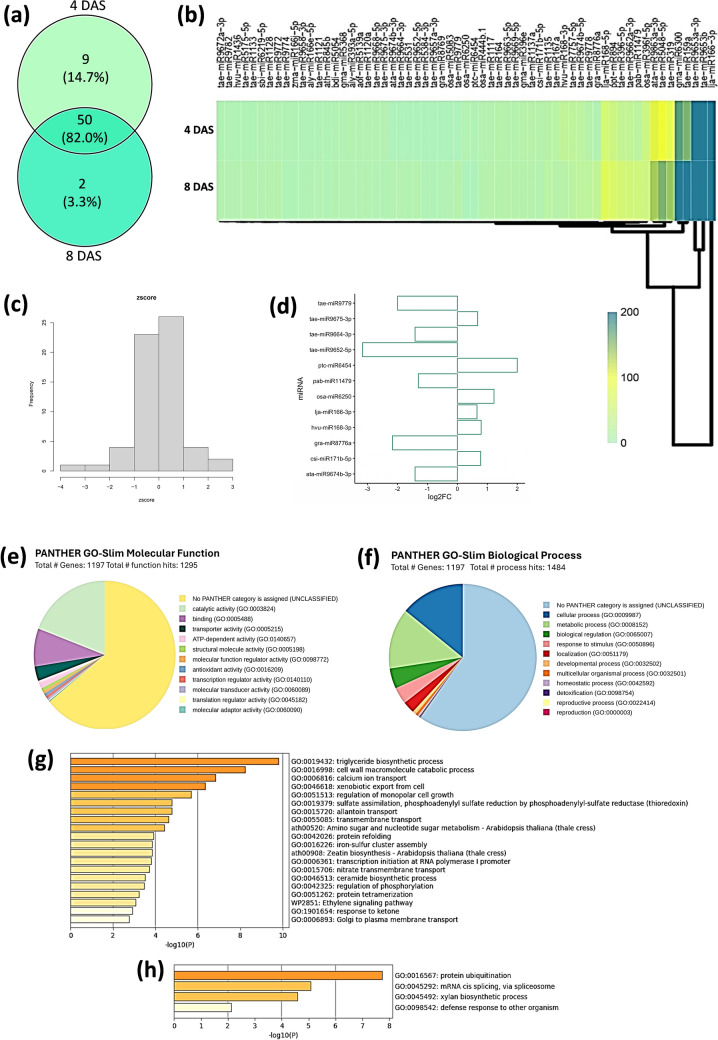
MiRNome analysis. **a** Venn diagram of miRNA percentage fractions expressed at 4 DAS, 8 DAS, and both conditions. **b** Heatmap showing the expression profile of the 61 miRNAs detected in *T. aestivum* seminal root at 4 and 8 DAS, together with the relative hierarchical clustering. **c** Distribution of the *Z*-score values calculated for the 61 miRNAs. **d** Bar plot showing the changes registered for the 12 differentially expressed miRNAs (DEMs; *Z*-score ≥ or ≤ 1) between 4 and 8 DAS in *T. aestivum* seminal roots. **e** PANTHER pie chart of enriched terms (relative to the category of molecular functions) based on the predicted targets for DEMs. **f** PANTHER pie chart of enriched terms (relative to the category of biological processes) based on the predicted targets for DEMs. **g** Metascape bar graph of top level GO enriched terms based on the predicted targets for DEMs (bar color indicates the *p*-value). **h** Metascape bar graph of top level GO enriched terms based on a subgroup of predicted targets for DEMs selected on expectation value (bar color indicates the *p*-value)

Recently, miRNAs have assumed particular importance due to their capability in carrying out post-transcriptional regulation phenomena of target genes implicated in root development (Couzigou & Combier 2016). Indeed, their expression is linked to different pathways controlling phytohormones signaling, nutritional metabolism, and signal transduction (Meng et al. 2010). In *A. thaliana* and in *Oryza sativa* L., it has been demonstrated that miRNAs participate in primary, lateral and adventitious root growth and vascularization, as well as in response to biotic and abiotic stressors (Khan et al. 2011; Yan et al. 2022). In particular, miRNAs seem to play a pivotal role also in embryonic root formation, considering that knock-out embryos for SERRATA, a protein involved in miRNAs biogenesis, show defects in the initiation of cotyledons and in the production of lateral organs (Prigge & Wagner 2001; Yang et al. 2006). Thus, to explore both the diversity and expression of miRNAs in wheat roots during the first weeks of growth, a miRNome analysis was performed through a small RNA sequencing approach. This analysis mapped almost 40 million reads across all the samples, identifying a total of 61 different sequences attributable to already known families of plant miRNAs (Supplemental Material – Table S3). The number of miRNAs expressed at 4 and 8 DAS is shown in Fig. 3a, together with the fraction co-expressed in both conditions. Based on the counts obtained, a heatmap relative to the abundance of each miRNA per sample was produced (Fig. 3b). From 4 to 8 DAS, only 14 miRNAs were upregulated. Meanwhile, 47 were downregulated. Most miRNAs were characteristic of the initial days of embryonic development. In fact, despite an 82% overlap between the two developmental stages, the downregulation of these key regulators at 8 DAS points to a strategic shift, potentially enabling the over-expression of mRNA targets aimed at driving tissue specialization. The transition from pluripotency to a differentiated state is governed by a dynamic interaction between transcriptional programs and epigenetic modifications. MiRNAs play an essential role within this network, fine-tuning the balance between self-renewal and lineage commitment. In this context, they modulate the expression of meristematic genes, coordinate cell proliferation, and activate differentiation-associated pathways (Vashisht & Nodine 2014). Even though their function during embryonic development is not yet fully understood, Plotiknova and colleagues (2019) have characterized dozens of miRNAs in *A. thaliana* embryos that seem to repress transcription factors required for a correct mitotic division, supporting the present data. For instance, it was found that the repression of TCP4 (TEOSINTE BRANCHED 1-CYCLOIDEA-PROLIFERATING CELL FACTOR 1/2 protein family, transcription factor 4) by miR319 is more active at the heart stage in the basal region of the embryo and is required for cotyledon formation. At the same time, miR165 and 166 are also abundant in those cells, repressing HD-Zip transcription factors essential for proper morphogenesis (Plotnikova et al. 2019). Moreover, during the early germination stage, miR156/157 activity has been shown to repress SPL transcription factor accumulation, preventing the expression of maturation phase genes (Nodine & Bartel 2010).

To compare miRNA expression levels between the two conditions, fold change and *Z*-score parameters were used. The first indicates the magnitude and direction of change, while the second evaluates if the expression change is significant. Choosing 1 as the *Z*-score threshold value (Fig. 3c), 12 microRNAs resulted to vary from 4 to 8 DAS in a significant way. In order to visualize the intensity and directionality of these differentially expressed miRNAs (DEMs), a bar plot was produced (Fig. 3d).

To understand the potential role of DEMs in wheat root development, the putative targets of these selected miRNAs were predicted. The list of all *T. aestivum* targets linked to the 12 DEMs was reported in full in Supplemental Material – Table S4. However, to make this information more easily available, only the targets showing low expectation values (that is high level of alignment between transcript and miRNA) are shown for each miRNA in Table 1.

**Table 1:** Targets predicted for the 12 DEMs. For each DEM (Query), the putative mRNA targets showing an expectation value (Exp) ≤ 1 (where possible, otherwise the lowest registered values)) were listed. For each target, IWGSC (Internation Wheat Genome Sequencing Consortium) ID (Accession) and full description (*Triticum* gene Description) were reported, as well as TAIR (The Arabidopsis Information Resource) ID (*Arabidopsis* ortholog Accession) and relative ortholog description (*Arabidopsis* gene description). The complete output of the bioinformatics analysis was reported in Supplemental Material—Table S4

Query	Exp	Target			
		Accession	*Triticum* gene description	*Arabidopsis* ortholog ID	*Arabidopsis* gene description
ata-miR9674b-3p	2.0	TraesCS2A02G318200.1	RING-type E3 ubiquitin transferase	AT1G22500	RING/U-box superfamily protein
				AT1G35330	
				AT1G72200	
				AT2G35000	
				AT3G05200	
				AT4G09100	
				AT2G34990	
				AT4G09120	
				AT4G09130	
				AT5G27420	
				AT4G09110	
csi-miR171b-5p	2.5	TraesCS6B02G470900.1	NAD-dependent epimerase/dehydratase domain-containing protein	AT2G21280	NAD(P)-binding Rossmann-fold superfamily protein
	2.5	TraesCS7D02G484800.1			
gra-miR8776a	1.0	TraesCS1A02G322300.1	Acylaminoacyl-peptidase	AT4G14570	Acylaminoacyl-peptidase-like protein
	1.0	TraesCS1B02G334600.1		AT4G14570	
	1.0	TraesCS1B02G334600.2		AT4G14570	
	1.0	TraesCS1D02G322400.1		AT4G14570	
	1.0	TraesCS1D02G322400.2		AT4G14570	
	1.0	TraesCS1D02G322400.3		AT4G14570	
	1.0	TraesCS1D02G322400.4		AT4G14570	
	1.0	TraesCS1D02G322400.5		AT4G14570	
	2.0	TraesCS7D02G258000.1	RIN4 pathogenic type III effector avirulence factor Avr cleavage site domain-containing protein	AT3G25070	RPM1 interacting protein 4
	2.0	TraesCS7D02G258000.2		AT3G25070	
hvu-miR168-3p	2.5	TraesCS4A02G269100.1	Protein kinase domain-containing protein	AT3G53640	Protein kinase superfamily protein
				AT3G25840	
				AT1G13350	
lja-miR166-3p	2.0	TraesCS1A02G157500.1	Uncharacterized protein	AT5G60690	Homeobox-leucine zipper family protein/lipid-binding START domain-containing protein
	2.0	TraesCS1B02G173900.1		AT5G60690	
	2.0	TraesCS1D02G155200.1	Homeobox domain-containing protein	AT5G60690	
	2.0	TraesCS1D02G155200.2	Homeobox domain-containing protein	AT5G60690	
	2.0	TraesCS1D02G155200.3	Uncharacterized protein	AT5G60690	
	2.0	TraesCS3A02G312800.1		AT1G52150	
	2.0	TraesCS3A02G312800.2		AT1G52150	
	2.0	TraesCS3B02G159100.1		AT1G52150	
	2.0	TraesCS3B02G159100.2		AT1G52150	
	2.0	TraesCS3D02G141500.1		AT1G52150	
	2.0	TraesCS3D02G141500.2		AT1G52150	
	2.0	TraesCS4B02G385200.1		AT5G60690	
	2.0	TraesCS4D02G359600.1		AT5G60690	
	2.0	TraesCS5A02G549700.1	START domain-containing protein	AT5G60690	
	2.0	TraesCS5A02G549700.2		AT5G60690	
osa-miR6250	2.0	TraesCS6B02G160200.1	Uncharacterized protein	AT2G15440	polysaccharide biosynthesis protein (DUF579)
				AT3G50220	
				AT5G67210	IRREGULAR XYLEM protein (DUF579)
pab-miR11479	2.0	TraesCS1D02G203000.1	PLAC8 family protein	AT4G23470	PLAC8 family protein
	2.0			AT1G63830	
	2.0			AT5G41390	
	2.0	TraesCS1D02G203000.2		AT1G63830	
	2.0			AT4G23470	
	2.0			AT5G41390	
	2.0	TraesCS2A02G553300.1	Uncharacterized protein	AT1G65730	YELLOW STRIPE like 7
	2.0	TraesCS3A02G053800.2		AT1G25570	Di-glucose binding protein with Leucine-rich repeat domain-containing protein
	2.0	TraesCS3B02G064800.2	Malectin-like domain-containing protein	AT1G25570	Di-glucose binding protein with Leucine-rich repeat domain-containing protein
	2.0	TraesCS3B02G064800.3			
ptc-miR6454	2.5	TraesCS4B02G221900.1	phosphomevalonate kinase	AT1G31910	GHMP kinase family protein
tae-miR9652-5p	1.5	TraesCS3B02G287600.1	Uncharacterized protein	AT2G27880	Argonaute family protein
		TraesCS3B02G287600.2			
		TraesCS5A02G446000.1			
		TraesCS5A02G446000.2			
		TraesCS5B02G451400.1			
		TraesCS5B02G451400.2			
		TraesCS5B02G452500.1			
		TraesCS5B02G454200.1	Protein argonaute MEL1		
		TraesCS5B02G454200.2			
		TraesCS5B02G455700.1			
		TraesCS5B02G458100.1			
		TraesCS5D02G192700.1	Uncharacterized protein		
		TraesCS5D02G192700.2			
		TraesCS5D02G454200.1			
		TraesCS5D02G454200.2			
		TraesCS5D02G454600.1	Piwi domain-containing protein		
tae-miR9664-3p	3.0	TraesCS1D02G186500.1	Trehalase	AT4G24040	trehalase 1
	3.0	TraesCS2B02G245100.1	WAT1-related protein	AT1G43650	nodulin MtN21/EamA-like transporter family protein
				AT5G64700	
	3.0	TraesCS4A02G223500.1	NB-ARC domain-containing protein	AT2G17440	plant intracellular ras group-related LRR 5
	3.0	TraesCS4D02G089400.1		AT4G35470	plant intracellular ras group-related LRR 4
tae-miR9675-3p	2.5	TraesCS1A02G086100.1	Carboxypeptidase	AT2G35770	serine carboxypeptidase-like 28
		TraesCS1A02G086100.2			
		TraesCS1B02G104500.1			
		TraesCS1D02G087600.1			
tae-miR9779	1.0	TraesCS3B02G260900.1	Mitogen-activated protein kinase	AT1G18150	Protein kinase superfamily protein
				AT1G73670	MAP kinase 15
				AT3G18040	MAP kinase 9
		TraesCS3D02G221700.1		AT1G18150	Protein kinase superfamily protein
				AT1G73670	MAP kinase 15
				AT3G18040	MAP kinase 9

MiRNA expression profiles and corresponding predicted targets indicated the regulation of several molecular processes, associated not only with root development and cell homeostatic mechanisms but also with the control and biogenesis of miRNAs themselves. For instance, a pronounced downregulation of miR9652 and a concurrent increase in miR168 levels were observed in wheat seminal roots between 4 and 8 DAS (Fig. 3d). Importantly, the miR168 detected in the wheat samples was identified as *H. vulgare* L. miR168 (hvu-miR168-3p, miRBase) as it is currently not annotated in *T. aestivum*. This opens significant perspectives, revealing the existence of a novel miRNA in this species that may play a key role. Indeed, bioinformatic analysis predicted for mi9652 and miR168 the mRNA targets codifying for an Argonaute (AGO) family protein and a superfamily kinase protein (in particular, Serine–Threonine protein kinase PRP4 homolog), respectively, both critically involved in the modulation of miRNA function and synthesis. Indeed, AGO proteins are the primary interactors of small RNAs in the assembly of RISCs, enabling the recognition of specific transcripts through sequence complementarity. These proteins are the main mediators of target cleavage and translational inhibition, as well as chromatin remodeling (Vaucheret 2008; Zhang et al. 2015). On the other side, the kinase activity of PRP4K and its homologs is associated with the activation of miRNA production. Specifically, hyper-phosphorylation of the SERRATA protein (SE) mediated by PRP4K and other kinases leads to SE degradation, whereas the hypo-phosphorylated form regulates miRNA transcription, splicing and processing (Kanno et al. 2018; Wang et al. 2022a, b). Thus, it is reasonable to hypothesize that the role of the two aforementioned miRNAs is essential for the control of the miRNA machinery during embryonic root development.

As previously suggested by transcriptomics data, miRNomics evidence also suggested a fine coordination of DNA replication and protein modification and degradation during the investigated phases of root development. The decreased expression of miR9674 and miR8776, which have been bioinformatically predicted to target a RING-type E3 ubiquitin ligase and an acylaminoacyl-peptidase-like protein in that order, might be linked to an increased activity of these regulatory mechanisms. Indeed, ubiquitin ligases participate in one of the major post-translational modification systems in eukaryotic cells (Zeng et al. 2008), attaching ubiquitin units to substrate proteins (Kraft et al. 2005). Ubiquitinome analysis in rice (*O*. *sativa* ssp. japonica, cv. Nipponbare) seedlings has documented how ubiquitination influences protein localization, activity, and degradation during the early stages of germination (Arc et al. 2011; Han et al. 2014). In a similar way, acylamino acid-releasing enzymes (AAREs) are serine proteases that maintain cellular proteostasis and metabolic balance due to their dual enzymatic activity as exopeptidases, removing N-terminally acetylated amino acids from peptides, and endopeptidases, degrading oxidized or glycated proteins. This versatility enables AAREs to participate in the clearance of damaged proteins, particularly those affected by reactive oxygen species (ROS), underscoring their importance in cellular detoxification processes (Hoernstein et al. 2025). Experimental evidence from knockout mutants in *A. thaliana* has demonstrated that the loss of AARE function leads to increased levels of oxidized proteins and altered developmental timing, highlighting the role of this class of enzymes in regulating life span and developmental transitions (Nakai et al. 2012). Thus, this information corroborates the present data being in line with them.

Transcriptomics revealed the existence of cell cycle checks consistent with cellular root development and growth, evidence which is supported also by miRNomics. The downregulation of miR9779 is an example, being its predicted targets the MAP kinases 15 and 9, that are members of a protein family known to play a central role in regulating cell division (Mishra et al. 2006). Additionally, the repression of miR11479, able to act on PLAC8 protein expression, was observed. Colle and colleagues (2011) have identified PLAC8 motif-containing proteins as components of the *A. thaliana* deathsome and as factors involved in programmed cell death. However, different research groups have associated PLAC8 proteins also with diverse physiological roles in plants, including regulation of organ size and cell proliferation, nodule formation, metal tolerance, and calcium signaling (Cabreira-Cagliari et al. 2018), suggesting the involvement of PLAC8s in development and stress responsive pathways.

The omics approaches converged in identifying the modulation of both primary and secondary metabolism, including cell wall biosynthesis and modification. In this regard, the bioinformatic prediction linked serine carboxypeptidase-like (SCPL) protein to miR9675, which was found to be upregulated. MiR9675 has been previously characterized in *T. aestivum* and associated with heat stress tolerance (Saroha et al. 2024), while SCPLs seems to participate in various biochemical functions, such as the modification of phytochemicals and the regulation of phytohormone pathways. Moreover, SCPLs are increasingly recognized for their involvement in plant defense responses (Fraser et al. 2005; Liu et al. 2022; He et al. 2024). Similar to miR9675, miR6250 appeared upregulated at 8 DAS, suggesting a broader contribution of these two miRNAs in coordinating root-specific responses, especially cell wall production. Indeed, miR6250, although ubiquitous, has been detected as particularly expressed in roots, especially under abiotic stress (Liu 2012; Secco et al. 2013). In addition, the target of miR6250 was identified as the transcript encoding the IRREGULAR XYLEM (IRX) protein, belonging to the DUF579 domain-containing protein family. In higher plants, DUF579 proteins are grouped into four clusters and those included in the Clade II, like IRX15 and IRX15L, are involved in xylan biosynthesis and its deposition in secondary cell wall. Therefore, considering that *irx15 irx15l* double mutants exhibit disrupted the secondary wall architecture and enhanced sugar release during saccharification (Brown et al. 2011; Temple et al. 2019; Li et al. 2024), the stability and/or the translational control of the mRNAs for these proteins could be essential for a correct root development, due to the importance of secondary cell wall plasticity during seeding growth.

The information gained by high-throughput screening indicated that gene regulation mechanisms during wheat root growth were also exerted at the transcriptional level. As shown in Fig. 3d, a decrease in the expression of miR166 and miR171 could be observed in the seminal roots at 8 DAS. These two microRNAs are known to be ubiquitous and conserved across various plant tissues and species. However, various studies have documented a correlation between their expression and apical and root meristematic activity, as well as their involvement in phytohormonal signaling (Liu et al. 2009; Singh et al. 2017; Han & Zhou 2022). Indeed, the predicted targets for these miRNAs are two transcription factors, namely a Homeobox-leucine zipper family protein/lipid-binding START domain-containing protein (*TraesCS1A02G157500*) and a NAD(P)-binding Rossmann-fold superfamily protein (*TraesCS1D02G030700*) respectively, which are known to be involved in root morphogenesis (Pysh et al. 1999; Bolle 2004; Zhang et al. 2012; Sessa et al. 2018).

Lastly, gene ontology enrichment analysis was conducted on the complete list of targets predicted for DEMs (Supplemental Material – Table S4), in order to understand the main cellular pathways activated in wheat root development and to validate the hypotheses formulated thus far (Fig. 3e-h). Regarding MF category, functional enrichment analysis carried out by PANTHER showed that the most significant terms were catalytic activity, binding, transport, structural molecule activity, molecular function regulators, and antioxidant activity (Fig. 3e). For the BP category, instead, the same web tool revealed a strong enrichment in classes named cellular processes, metabolic processes, biological regulation, response to *stimulus*, localization, and developmental processes (Fig. 3f). In parallel, the analysis performed with Metascape highlighted a broader and more specific group of functional categories, such as macromolecule catabolic processes related to cell wall components, regulation of cell growth, sulfate assimilation, amino sugar and nucleotide sugar metabolism, and protein refolding (Fig. 3g). To increase the significance of the results, the same analysis was carried out via Metascape also on the subset of targets for DEMs filtered on the basis of the expectation value (Table 1). In this case, molecular processes associated with ubiquitination, mRNA splicing, xylan biosynthetic process, and plant response and defense mechanisms were identified (Fig. 3h).

### MiR166, miR168 and miR171 content significantly changes during root development, together with phytohormonal levels

Overall, both omics approaches consistently identified the main biological mechanisms governing wheat seminal root development, notably: miRNA regulation; phytohormone biosynthesis and signaling; cell wall formation. Thus, in the last part of the present research, the attention was directed toward three miRNAs identified as DEMs through the miRNome profiling and potentially linked with these molecular processes: miR168, miR166, and miR171.

Previously, Li and colleagues (2013) have identified these three microRNAs in *T. aestivum* seedlings through integrated miRNomics and degradome analyses conducted seven days after germination. The widespread conservation of these miRNAs across plant species is noteworthy, suggesting a fundamental role maintained through evolution. Taking this into account, the expression levels of the selected miRNAs were checked further by RT-qPCR, together with those of their respective targets and correlated genes, to corroborate the miRNomics data and to validate the bioinformatic predictions. These investigations were carried out on wheat seminal root samples collected at 4, 8, and even 12 DAS; the latter experimental point was added to assess whether the observed trends remained constant during the second week of development.

**Fig. 4:**
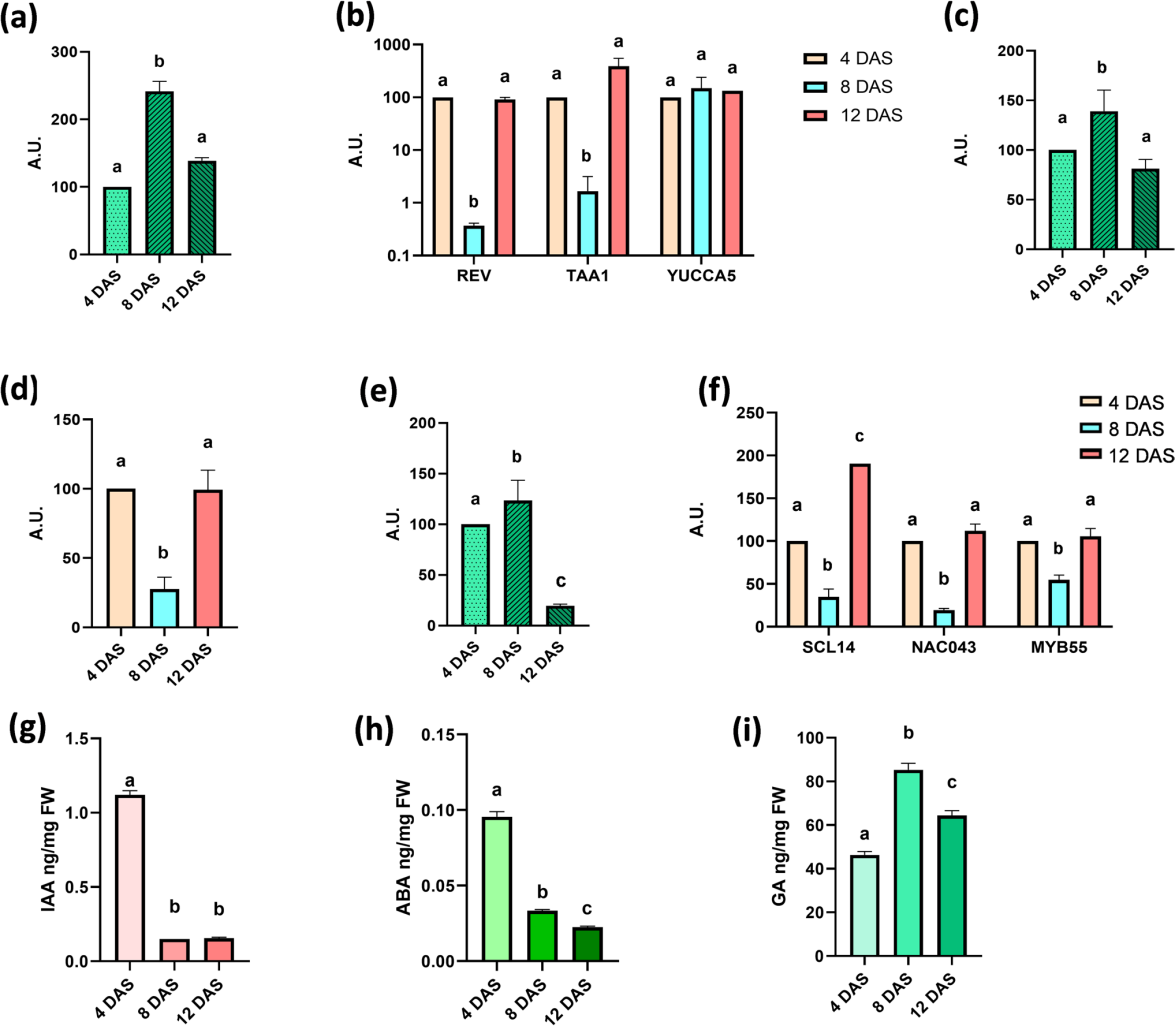
Gene expression analysis and liquid chromatographic results. Graphical representation of RT-qPCR data relative to the expression levels of: **a** miR166; **b** *Revoluta* (REV), *L-tryptophan–pyruvate aminotransferase 1* (TAA1) and *indole-3-pyruvate monooxygenase YUCCA5* (YUCCA5); **c** miR168; **d** *Serine/threonine protein kinase PRP4 homolog* (PRP4KA); **e** miR171; **f** *Scarecrow-like protein 14* (SCL14), *NAC domain-containing protein 43* (NAC043) and *Transcription factor MYB55* (MYB55). MiRNA levels were normalized with respect to 5S, while mRNA levels to *eIF4A*. All results were expressed in arbitrary units (A.U.) compared to the 4 DAS, considered as 100. Quantitation data of indole-3-acetic acid (IAA) **g** abscisic acid (ABA) **h** and GA **i** measured by liquid chromatographic approach. Phytohormone levels were expressed as ng per mg of plant material. All data were shown as mean ± standard deviation of three independent biological replicates. Different letters mean significant differences among samples at *p-*value ≤ 0.05

Numerous studies have highlighted the role of miR166, in concert with miR165, in root system development. These miRNAs are activated by transcription factors, such as SHORT ROOT (SHR) and SCARECROW (SCR), whose expression is crucial not only for vascular patterning but also for root architecture, by targeting in turn the HD-ZIP III transcription factors REVOLUTA and PHABULOSA, respectively (Carlsbecker et al. 2010; Miyashima et al. 2011). Wei et al. (2023) have demonstrated that knockdown of miR166, and the consequent over-expression of its target gene, leads to inhibition of vascular development and cell wall formation. Moreover, miR166 seems to be implicated in the regulation of several hormonal pathways (Singh et al. 2017). For instance, in maize, miR166 inactivation would lead to increased levels of abscisic acid (ABA) and decreased levels of indole-3-acetic acid (IAA) (Li et al. 2020a, b). In fact, REVOLUTA, a known target of miR166, acts as a transcriptional activator of various genes, including TRYPTOPHAN AMINOTRANSFERASE OF ARABIDOPSIS 1 (TAA1) and YUCCA5 (YUC5) which are involved in auxin biosynthesis (Yan et al. 2017). Analysis of miR166 expression by qPCR revealed a trend consistent with the miRNome data, showing an increase at 8 days of growth; however, at 12 DAS this miRNA returned to the level registered at 4 DAS (Fig. 4a). On the other hand, REVOLUTA (REV) and TAA1 displayed an expression pattern opposite to that of miR166, as expected for its targets. By contrast, YUCCA 5 did not exhibit any significant variation over time (Fig. 4b).

MiR168 has been associated with plant responses to various environmental stressors, such as salinity (Sunkar et al. 2007). In maize and wheat, high salt conditions have been reported to alter the expression of this miRNA, while in rice its silencing under the same conditions resulted in enhanced plant growth and increased root length (Ding et al. 2009; Tang et al. 2012; Eren et al. 2015). Interestingly, unlike other plant species where AGO1, an RNA silencer protein, has been identified as the canonical target of miR168 (Qi et al. 2005), in the wheat Altamira variety, as documented in this study, the target is PRPKA, a protein involved in the regulation of miRNA splicing and thus in their biogenesis and functional modulation. qPCR assays confirmed that miR168 increased at 8 DAS, in line with miRNomics, and then diminished at 12 DAS, reaching the amount detected at 4 DAS (Fig. 4c). Also in this case, the target PRPK4A exhibited an inverse trend to its corresponding miRNA. In detail, the transcript levels decreased from 4 to 8 DAS (− 72%; *p* ≤ 0.05) and then increased from 8 to 12 DAS (+ 259%; *p* ≤ 0.05) (Fig. 4d).

Similar to the other two miRNAs, miR171 has been recognized as highly conserved across a wide range of species and involved in plant growth and development, as well as in responses to both abiotic and biotic stresses (Pei et al. 2023). MiR171c over-expression in *A. thaliana* has been shown to alter growth not only in the apical region but also in the root system (Wang et al. 2010; Xue et al. 2014). This is because most of its targets belong to the GRAS (GAI-RGA-SCR) gene family. GRAS genes encode a broad class of transcription factors characterized by a conserved C-terminal GRAS domain. They are widely distributed throughout the plant kingdom and play crucial roles in signal transduction and stress responses (Jaiswal et al. 2022; Waseem et al. 2022). The GRAS family has been subdivided into several clades, based on shared structural and functional features, including the DELLA, SCR, Ls, HAM, PAT1, SHR, and SCL9 branches. The latter includes several members, such as SCL9, SCL33, SCL31, and SCL14, but their biological activities remain still relatively poorly understood (Bolle 2004). In particular, SCL14, a member of this group, resulted as one of the putative targets for miR171 by the present bioinformatic predictions. In this regard, Chen et al. (2015) have reported that SCL14 is expressed in various tissues of *T. aestivum*, although predominantly in roots, and its silencing leads to a significant reduction in both root weight and length. This morphological alteration may be explained considering that in *Populus* L. it has been proved that SCL14 is implicated in lignin biosynthesis and secondary cell wall formation. Specifically, under low gibberellic acid (GA) conditions, SCL14 interacts directly with the transcription factor NAC043, suppressing the downstream transcription of MYB61 (ortholog of the MYB55 wheat gene) and thereby inhibiting lignin biosynthesis (Wu et al. 2023). qPCR data for miR171 content perfectly fitted with those from miRNomics: an increase in expression was found at 8 DAS, followed by a significant decrease at 12 DAS (− 80%, *p* < 0.01) (Fig. 4e). In addition, all miR171 predicted targets, namely SCL14, NAC043, and MYB55, exhibited an opposite expression trend, as expected (Fig. 4f). In more detail, all target profiles showed a perfect overlap with that of miR171, except for NAC043 and MYB55, which were less responsive at 12 DAS.

Taken together, these findings confirmed that miR166, miR168, and miR171 exhibit a peak of expression in wheat seminal roots at 8 DAS. Then, their levels appear reduced at 12 DAS, returning to the levels measured at 4 DAS or even lower (in the case of miR171). This study also validates the bioinformatic target predictions for these three miRNAs, highlighting their key role in *T. aestivum* root development. Plant hormones, which play a major role in growth and acclimation, interact closely with miRNA pathways. In particular, this crosstalk contributes significantly to the modulation of root development and adaptive mechanisms to environmental stimuli (Gray 2004; Jones-Rhoades et al. 2006; Liu & Chen 2009; Singh et al. 2021). One of the earliest pieces of evidence for this link was provided by Han and colleagues (2004), who have observed that *hyponastic leaves 1* mutant (defective in miRNA biogenesis) of *Arabidopsis* plants exhibited altered responses to various phytohormones, including ABA, cytokinins and auxin. In this regard, numerous spatial and temporal expression patterns of various miRNAs are known to be influenced by cis-regulatory elements, such as phytohormones (Singh et al. 2017). For this reason, to provide a wider characterization of wheat roots at the selected growth stages, the levels of IAA, ABA, and GA were assessed by liquid chromatographic analysis (Fig. 4g-i). The results revealed that during the first week of growth (i.e., from 4 to 8 DAS) a significant decrease of IAA (*p* < 0.001; 4 DAS: 1.12 ng/mg FW; 8 DAS: 0.15 ng/mg FW) and ABA (*p* < 0.01; 4 DAS: 0.095 ng/mg FW; 8 DAS: 0.033 ng/mg FW) could be observed. By contrast, GA levels showed a relevant increase (+ 85%) during the same period (*p* < 0.01; 4 DAS: 46.25 ng/mg FW; 8 DAS: 87,39 ng/mg FW). From 8 to 12 DAS, IAA remained constant, while ABA continued to decline significantly (*p* < 0.01; 12 DAS: 0,022 ng/mg FW). Similarly, GA exhibited a slight but substantial reduction (*p* ≤ 0.05; 12 DAS: 65,98 ng/mg FW), without reaching the level detected at 4 DAS.

The relevance of ABA and IAA in the growth of both aerial and root tissues is nowadays well established (Walton 1980; Casimiro et al. 2003). Thus, their biosynthesis, concentration in meristematic regions, such as root tips, and transport are tightly regulated.

IAA plays a central role in the development of all plant organs (Garay-Arroyo et al. 2012). Synthesized primarily in young leaves and shoot apical meristems, it is moved in the roots via the phloem; anyway, it can also be produced locally in small amounts, participating in the generation of auxin gradients necessary to maintain root meristem activity (Ljung et al. 2001; Ljung et al. 2005; Ikeda et al. 2009; Petterson et al. 2009). High auxin levels have been predominantly detected in the quiescent center, where mitotic activity is minimal, while in the surrounding meristematic region the lower concentrations correlate with rapid cell differentiation processes (Grieneisen et al. 2007). On the other hand, ABA is an isoprenoid phytohormone involved not only in seed development and dormancy but also in the root response to environmental *stimuli*, such as drought and salt stress (Finkelstein et al. 2002; Zhang et al. 2006; Daszkowska-Golec 2016). The spatial distribution of ABA is also critical for the morphological organization of root growth regions. In detail, ABA accumulation at the root apex contributes to the maintenance of the quiescent center and suppresses differentiation phenomena within the stem cell niche. In fact, mutants defective in ABA biosynthesis exhibit premature differentiation of stem cells (Zhang et al. 2010). In other regions, such as endodermis, ABA levels are maintained low and stable to support proper cell elongation events: for example, exogenous application of ABA at high concentrations (1 µM) has been shown to inhibit root growth (Ghassemian et al. 2000), while *scr1* mutants (lacking for the SCR protein that is a repressor of ABI4, a transcription factor activated by ABA signaling) exhibit shorter roots (Cui et al. 2012). According to all these premises, the significant decrease in IAA (− 84%) and ABA (− 97%) levels observed between 4 and 12 DAS may reflect their specific roles in root development during early growth stages. Indeed, in support of the concepts mentioned above, Rock and Sun (2005) have demonstrated that IAA has an endogenous role in cell elongation, pattern formation, and differentiation, while ABA mainly regulates lateral root development and the balance between dormancy and germination. During seed germination, auxin and ABA pathways interact to control radicle protrusion, with auxin promoting embryonic root emergence through ARF-mediated signaling and ABA maintaining dormancy (Liu et al. 2013). Furthermore, auxin plays a critical role in establishing root apical meristem organization, particularly in defining the quiescent center and columella cell pattern, through the AUX1/LAX family of auxin influx carriers (Ugartechea-Chirino et al. 2009). Given the importance of auxin in root apical meristem patterning and the fact that our hormone measurements were conducted on whole root systems, we hypothesize that the observed decline in IAA levels may not necessarily indicate an absolute reduction in auxin content, but rather a specific and focused redistribution of this hormone along the developing root axis, to establish proper spatial gradients required for meristem organization and radial patterning. Therefore, it is plausible that the decline of these hormones is associated with the need to promote cell division rather than differentiation. In parallel, GA concentration was higher compared to IAA and ABA at all sampling times. Although, high levels of GA in roots have already been demonstrated by Yaxley et al. (2001), this observation can be explained by the pivotal role of this phytohormone in regulating seed germination and root growth (Pacifici et al. 2015). Indeed, in roots, gibberellins are essential for the maintenance and development of the root meristem along both radial and longitudinal axes (Shtin et al. 2022). In *Arabidopsis* roots, it has been shown that during the early stages of germination (between 3 and 5 DAS), elevated doses of GA in roots promote cell division, repressing cytokinin signaling and inhibiting middle cortex formation. Around 8 DAS, the increased expression of the HOMEODOMAIN-LEUCINE ZIPPER III (HD-ZIPIII) transcription factor PHABULOSA (PHB), partly driven by a reduction in the expression of the miR165-166 axis (as demonstrated previously by omics), has been linked to gibberellin catabolism, thereby promoting cell differentiation and contributing to the control of root meristem size (Paquette & Benfey 2005; Bertolotti et al. 2021a, 2021b). This result would justify the observed reduction of GA at 12 DAS compared to 8 DAS.

## Conclusion

In light of the increasing global demand for wheat and the challenges posed by the current climate change, improving early root development represents a strategic avenue to enhance crop resilience and productivity. The seminal root system plays a pivotal role in seedling establishment, nutrient uptake, and overall plant vigor. However, the molecular mechanisms governing its development during the first weeks of growth remain largely unexplored in *T. aestivum*. This knowledge gap is particularly critical given that roots are highly sensitive to environmental fluctuations, such as temperature extremes and soil water availability, which increasingly threaten wheat cultivation. Although breeding programs have targeted root features through Quantitative Trait *Loci* mapping and marker-assisted selection, the dynamic nature of ecological conditions limits the universal adaptability of selected genotypes. Recent scientific advances highlight the potential role of miRNAs as key post-transcriptional regulators of root development, offering promising molecular targets to improve seminal root performance, even under stress. Thus, a deeper understanding of these regulatory networks during the initial growth stages of sprouts is essential to unlock new strategies for optimizing wheat productivity and quality, toward a model of smart agriculture based on sustainability in a changing climate.

Through an integrated transcriptomic and miRNomic approach, this study provides a comprehensive overview of the gene expression dynamics and miRNA-mediated regulation in *T. aestivum* seminal roots. The results revealed a coordinated activation of genes involved in cell division, metabolic reprogramming, and hormonal signaling during the first weeks of growth. Our findings also open new perspectives on the involvement of some specific miRNAs, such as miR166, miR168, and miR171, in wheat root growth, with expression patterns inversely correlated to their respective transcript targets. Despite these preliminary insights, the molecular roles of these and other wheat root miRNAs remain largely under-investigated. Therefore, the present research represents a fundamental contribution to future studies in this direction. Anyway, in the present work, the mRNA targets for all miRNAs detected in wheat seminal roots were predicted and some of them, those relative to miR166, miR168, and miR171, were validated by qPCR assays. Lastly, a refined temporal analysis of indole-3-acetic acid, abscisic acid, and gibberellic acid was carried out in wheat roots using chromatographic approach, confirming the existence of a finely regulated hormonal production partially modulated by miRNA activities. Taken together, these findings underscore the potential involvement of miRNAs in orchestrating early root development, integrating transcriptional, post-transcriptional, and metabolic regulatory layers essential for balancing cell division and differentiation. These insights not only deepen our understanding of wheat root biology under normal conditions but also open promising perspectives for biotechnological applications. Future research may exploit the manipulation of miRNAs to engineer wheat varieties with enhanced root systems, developing novel strategies to improve crop growth, yield and quality under variable environmental conditions.

## Supplementary Information

Below is the link to the electronic supplementary material.

Supplementary file1 (DOCX 19 KB)

Supplementary file2 (XLSX 76 KB)

Supplementary file3 (XLSX 13 KB)

Supplementary file4 (XLSX 330 KB)

## Data Availability

Data are available within the article and its supplementary files. Sequencing data are available at NCBI BioProject database (BioProject ID: PRJNA1438249).
