# Supplementary material for: High-throughput sequencing reveals that microRNA-based regulation, cell wall remodeling and phytohormone signaling orchestrate wheat seminal root development
Source: Planta. 2026 Jul 6;264(2):46. doi: 10.1007/s00425-026-05069-w (PMC13337622; doi:10.1007/s00425-026-05069-w)
Supplement: Supplementary file 1 — Supplementary file1 (DOCX 19 KB) [file 425_2026_5069_MOESM1_ESM.docx]

**SUPPLEMENTAL MATERIAL**

**Supplemental Material - Table S1.** Primers used in RT-qPCR analyses. PrimerBLAST software was used to develop *Triticum aestivum*-specific primers (selected parameters: min total mismatches = 1; min 3’ end mismatches = 1; PCR product size = 70-160; optimal primer size = 20; optimal Tm = 60).

|  | | | |
| --- | --- | --- | --- |
| Gene name | Primer Forward 5’-3’ | Primer Reverse 5’-3’ | Reference |
| REVOLUTA | CCAAGCTGTGAATCTGTGGTC | CGATCTTTGAGGATCTCTGCA | Liu *et al*., 2014 |
| TAA1 | GCCACGCAAACGCAGGGTAAG | ATCCGGGTAGTCCCTCCGGTT | Yan *et al.,* 2017 |
| YUCCA5 | TCGTCCCGGGAATTAAACGGTTCT | CCGATTTCCCTTTCCACGCGTTT | Challa *et al.,* 2016 |
| PRP4K1A | ATGGCATGCTTTGGCTTCAC | AAAGGCTCCCTTTCGAAGCA | PrimerBLAST (Ye *et al.,* 2012) |
| SCL14 | CGTGGACGATAGTGAGATGGT | TGCTGCCTGATTCGCTTCA | Chen *et al.,* 2015 |
| NAC043 | TATACCCCCTTCCCCAACCC | GAACTGGGGAGAGATGCCAA | PrimerBLAST (Ye *et al.,* 2012) |
| MYB55 | TCCAGAAGTCACTCGGCAAC | GATCTCGTTGTCCGTCCTCC | PrimerBLAST (Ye *et al.,* 2012) |
| eIF4A | TCATAGATCTGGTCCTTGAAACC | GGCAGTCTCTTCGTGCTGAC | Li *et al.*, 2015 |

**References**

Challa, K.R., Aggarwal, P. and Nath, U. (2016) Activation of YUCCA5 by the transcription factor TCP4 integrates developmental and environmental signals to promote hypocotyl elongation in *Arabidopsis*. *Plant Cell*, **28**, 2117–2130. <https://doi.org/10.1105/tpc.16.00360>

Chen, K., Li, H., Chen, Y., Zheng, Q., Li, B. and Li, Z. (2015) TaSCL14, a novel wheat (*Triticum aestivum* L.) GRAS gene, regulates plant growth, photosynthesis, tolerance to photooxidative stress, and senescence. *Journal of Genetics and Genomics*, **42**, 21–32. <https://doi.org/10.1016/j.jgg.2014.11.002>

Li, J., Xu, H.H., Liu, W.C., Zhang, X.W. and Lu, Y.T. (2015) Ethylene inhibits root elongation during alkaline stress through AUXIN1 and associated changes in auxin accumulation. *Plant Physiology*, **168**, 1777–1791. <https://doi.org/10.1104/pp.15.00523>

Liu, Y., You, S., Taylor-Teeples, M., Li, W.L., Schuetz, M., Brady, S.M. and Douglas, C.J. (2014) BEL1-LIKE HOMEODOMAIN6 and KNOTTED *ARABIDOPSIS THALIANA*7 interact and regulate secondary cell wall formation via repression of REVOLUTA. *Plant Cell*, **26**, 4843–4861. <https://doi.org/10.1105/tpc.114.128322>

Yan, Z., Liu, X., Ljung, K., Li, S., Zhao, W., Yang, F., Wang, M. and Tao, Y. (2017) Type B response regulators act as central integrators in transcriptional control of the auxin biosynthesis enzyme TAA1. *Plant Physiology*, **175**, 1438–1454. <https://doi.org/10.1104/pp.17.00878>

Ye, J., Coulouris, G., Zaretskaya, I., Cutcutache, I., Rozen, S. and Madden, T.L. (2012) Primer-BLAST: a tool to design target-specific primers for polymerase chain reaction. *BMC Bioinformatics*, **13**, 1–11. <https://doi.org/10.1186/1471-2105-13-134>
